# Is the relationship between deprivation and outcomes in rheumatoid arthritis mediated by body mass index? A longitudinal cohort study

**DOI:** 10.1093/rheumatology/keac662

**Published:** 2022-11-28

**Authors:** Rozemarijn Witkam, James M Gwinnutt, Jennifer Humphreys, Suzanne M M Verstappen, RAMS Co-Investigators, Ade Adebajo, Khalid Ahmed, Atheer Al-Ansari, Roshan Amarasena, Marwan Bukhari, Margaret Callan, Easwaradhas G Chelliah, Hector Chinoy, Annie Cooper, Bhaskar Dasgupta, Martin Davis, James Galloway, Andrew Gough, Michael Green, Nicola Gullick, Jennifer Hamilton, Waji Hassan, Samantha Hider, Kimme Hyrich, Sanjeet Kamath, Susan Knight, Suzanne Lane, Martin Lee, Sarah Levy, Lizzy Macphie, Christopher Marguerie, Tarnya Marshall, Catherine Mathews, Frank McKenna, Sophia Naz, Mark Perry, Louise Pollard, Brian Quilty, Lindsay Robertson, Dipak Roy, Paul Sanders, Vadivelu Saravanan, David Scott, Gillian Smith, Richard Smith, Deborah Symmons, Lee-Suan Teh, Nick Viner

**Affiliations:** Centre for Epidemiology Versus Arthritis, Division of Musculoskeletal and Dermatological Sciences, The University of Manchester, Manchester, UK; Centre for Epidemiology Versus Arthritis, Division of Musculoskeletal and Dermatological Sciences, The University of Manchester, Manchester, UK; Centre for Epidemiology Versus Arthritis, Division of Musculoskeletal and Dermatological Sciences, The University of Manchester, Manchester, UK; NIHR Manchester Biomedical Research Centre, Manchester University NHS Foundation Trust, Manchester Academic Health Science Centre, Manchester, UK; Centre for Epidemiology Versus Arthritis, Division of Musculoskeletal and Dermatological Sciences, The University of Manchester, Manchester, UK; NIHR Manchester Biomedical Research Centre, Manchester University NHS Foundation Trust, Manchester Academic Health Science Centre, Manchester, UK

**Keywords:** socioeconomic position, obesity, RA, cohort study

## Abstract

**Objectives:**

To understand the relationships between deprivation and obesity with self-reported disability and disease activity in people with RA, and to determine whether BMI mediates the relationship between area-level deprivation and these outcomes.

**Methods:**

Data came from the Rheumatoid Arthritis Medication Study (RAMS), a 1-year multicentre prospective observational cohort of people with RA recruited from rheumatology centres across England commencing MTX for the first time. A total of 1529 and 1626 people were included who had a baseline and at least one follow-up measurement at 6 or 12 months of HAQ—Disability Index (HAQ-DI) and DAS in 28 joints (DAS28), respectively. Linear mixed models estimated the associations of deprivation and obesity with repeated measures HAQ-DI and DAS28. Causal mediation analyses estimated the mediating effect of BMI on the relationship between deprivation and RA outcomes.

**Results:**

Higher deprivation and obesity were associated with higher disability [adjusted regression coefficients highest *vs* lowest deprivation fifths 0.32 (95% CI 0.19, 0.45); obesity *vs* no obesity 0.13 (95% CI 0.06, 0.20)] and higher disease activity [adjusted regression coefficients highest *vs* lowest deprivation fifths 0.34 (95% CI 0.11, 0.58); obesity *vs* no obesity 0.17 (95% CI 0.04, 0.31)]. BMI mediated part of the association between higher deprivation and self-reported disability (14.24%) and DAS (17.26%).

**Conclusions:**

People with RA living in deprived areas have a higher burden of disease, which is partly mediated through obesity. Weight-loss strategies in RA could be better targeted towards those living in deprived areas.

## Graphical Abstract

**Figure:**
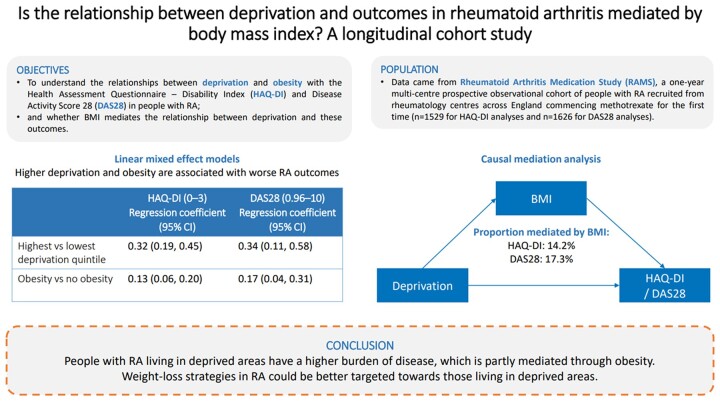

Rheumatology key messages

- Both deprivation and obesity were associated with worse disability and disease activity.
- BMI mediated 14.24–17.26% of the associations of deprivation with disability and disease activity.

## Introduction

RA is a progressive degenerative autoimmune disease, which if untreated can result in painful, swollen joints, severe disability and premature mortality [1]. Understanding risk factors associated with these poor outcomes in people with RA is important. If risk factors are modifiable, they can be targeted early in the disease process and if they are not easily modified, those most at risk for severe disease can be closely monitored by their clinicians.

Evidence from cross-sectional [2–5] and longitudinal [6–9] studies suggests that there are socioeconomic disparities in outcomes for people with RA. In order to address the worse disease outcomes among those from with lower socioeconomic position (SEP), it is important to understand why these discrepancies exist. The relationship between lower SEP and RA outcomes is likely (at least partly) indirect, with SEP influencing other intermediary factors, such as lifestyle and environmental factors, which in turn influence disability and disease activity. Understanding which factors mediate the relationship between lower SEP and RA outcomes may help to identify targets for intervention strategies.

A potential mediator for the relationship between a lower SEP and RA outcomes is obesity. Obesity rates are rising worldwide. In the UK, the latest estimates suggest that the majority of the adult population aged ≥16 years [68% (95% CI 66%, 70%) for men and 60% (95% CI 59%, 62%) for women] was either overweight or obese [10]. It is well-known that obesity is socially patterned: those with lower SEP are more likely to be obese [11]. Recent research also suggests a relationship between obesity and worse disability and disease activity [12–17] and a reduced chance of achieving remission in obese people with RA [12], potentially through the accumulation of pro-inflammatory cytokines in adipose tissue [18]. However, most of these studies did not adjust for socioeconomic factors and failed to acknowledge the complex interaction of SEP and obesity with RA outcomes.

As previous literature has suggested that both SEP and obesity increase the risk for worse outcomes in RA [2–9, 12–16], it is of clinical importance to understand how these factors interact. We hypothesized that obesity is a mediator for the relationship between deprivation and worse disease outcomes in RA; however, this has not yet been investigated. Therefore, this study aimed to understand (i) the relationships between area-level deprivation and disability and disease activity, separately; (ii) the relationships between obesity and disability and disease activity; and (iii) the mediating effect of BMI on the relationship between area-level deprivation and disability and disease activity in people with RA.

## Methods

### Study population

Data came from the Rheumatoid Arthritis Medication Study (RAMS), a 1-year prospective observational cohort of people with RA recruited between August 2008 and July 2019 from 38 rheumatology centres across England, who were about to start MTX for the first time. Inclusion criteria for RAMS were: being 18 years or older, having a medical diagnosis of RA and about to start MTX (either as monotherapy or combined with other conventional synthetic DMARDs) for the first time. Participants were excluded if they previously used biological DMARDs. Baseline assessment was just before participants started MTX and follow-up assessments were at 6 and 12 months after commencing MTX.

Participants were included for this study if they either had a HAQ—Disability Index (HAQ-DI) or DAS in 28 joints (DAS28) available at baseline and at least one follow-up (at either 6 or 12 months) and weight and height were measured at baseline to calculate BMI. Written informed consent was acquired from all participants. Ethical approval was obtained from Central Manchester Research Ethics Committee (REC number 08/H1008/25).

### Measurements

Data were obtained by a research nurse interviewing the participant [using case report forms (CRF)], patient questionnaires and by extracting information from participants’ clinical records. The patient questionnaires were sent to the co-ordinating centre in Manchester in a pre-paid envelope by either the study nurse or participants for entry into a secure database; however, both the CRF and information from clinical records were entered in the database locally by a study nurse.

### Exposure variables

Height and weight were self-reported in the CRF at baseline, at 6 and 12 months. BMI was then calculated by dividing each participant’s weight in kilograms by their height in metres squared (kg/m^2^). Obesity was defined as having a BMI of 30 kg/m^2^ or more.

Area-level deprivation was used as a proxy for SEP, and was measured using the Index of Multiple Deprivation (IMD) fifths. Using the participants’ postcode at baseline, the most recent IMD calculation (2010, 2015 or 2019) was used after participants’ baseline date. The IMD is a measure of small-area deprivation in England based on seven indicators of deprivation (income; employment; education, skills and training; health deprivation and disability; crime; barriers to housing and services; living environment) [19].

### Outcome variables

At baseline, 6 months and 12 months, participants completed the HAQ-DI in the patient questionnaire, which measures self-reported disability [20]. DAS28 was also calculated at baseline, 6 months and 12 months, incorporating information regarding the number of tender joints out of 28 joints, the number of swollen joints out of 28 joints and self-reported general wellbeing using the visual analogue scale (VAS) (0–100 mm, where 100 is the worst score) recorded in the CRF during the visit to the research nurse [21]. Blood samples for the measurement of CRP (mg/l) to measure inflammation were taken and sent to the UK Biobank, Stockport, UK. If blood samples were not available, CRP levels were taken from participants’ clinical records.

### Covariates/additional variables

Demographic and lifestyle covariates were recorded at baseline. Covariates relevant to this study included: age, gender, ethnicity (white, non-white) smoking status (never, current, ex-smoker), alcohol intake (yes/no) and physical activity (compared with people your own age—much more, more, the same, less, much less). Additional clinical variables included the ACR 1987 criteria [22], symptom duration (years), MTX starting dose (mg/week) and history of comorbidities from a predefined table (hypertension, diabetes, cardiovascular disease, asthma, chronic obstructive pulmonary disease, peptic ulcer disease, liver disease, renal disease, depression and cancer) (categorized into: no comorbidities, one comorbidity, two or more comorbidities). All variables were captured in the CRF, except for physical activity which was recorded in the patient questionnaire.

### Statistical analysis

Baseline characteristics of the study sample were reported for categorical and continuous data using frequencies (%) and means with s.d., respectively.

Linear mixed models (LMM) were used to estimate longitudinal associations between IMD fifths (reference group: least deprived fifth) and repeated measures of HAQ-DI and DAS28 (adjusted for age and gender) and between obesity (reference group no obesity) and repeated measures of HAQ-DI and DAS28 (adjusted for age, ethnicity, IMD, smoking, physical activity and alcohol consumption). As a sensitivity analysis, we also investigated the four separate components of the DAS28 (i.e. tender joints, swollen joints, inflammation level and VAS wellbeing score). Mixed models incorporate both fixed and random-effects, taking into account the correlation between an individual’s repeated measures. To investigate whether associations differed for subgroups (i.e. by gender, obesity status or IMD group), interaction terms between (i) IMD and gender, (ii) IMD and obesity and (iii) obesity and gender were included in the models. Where meaningful interaction effects were identified from inspection of the *P*-values of interaction terms, subgroup analyses were performed. As some of the exposure variables and covariates had missing data (all <5.5%), multiple imputation using chained equation was performed with 10 cycles [23]. These analyses were performed using Stata v14.

The mediating effect of BMI on the relationship between deprivation and HAQ-DI/DAS28 was estimated using the Causal Mediation Analysis package in R [24]. This method uses a counterfactual approach, and assigns all participants first as exposed and then unexposed to the exposure variable (e.g. deprivation). The causal total (i.e. total effect of deprivation on HAQ-DI/DAS28), indirect (i.e. the effect mediated by BMI) and direct (i.e. effect not explained by BMI) effects are then defined as the difference between the two potential outcomes [25, 26]. Listwise deletion was used to deal with missing data in the mediation analyses. Sensitivity analyses were performed to test exposure–mediator interaction and the assumption of sequential ignorability (i.e. the degree of unmeasured confounding) [26].

## Results

### Description of the cohort

Of the 2431 people consenting to RAMS with a baseline record, 1641 and 1770 had HAQ-DI and DAS28 scores at baseline with at least one follow-up at 6 or 12 months, respectively. After excluding those with missing (110 for HAQ-DI sample; 140 for DAS28 sample) or extreme BMI values (BMI <12 or BMI >60) (2 for HAQ-DI sample; 4 for DAS28 sample), the final samples comprised 1529 people for the HAQ-DI analyses and 1626 for the DAS28 analyses (Supplementary Fig. S1, available at *Rheumatology* online). The sample characteristics for the HAQ-DI and DAS28 were similar. For the HAQ-DI and DAS28 samples, respectively, the majority were female (67.0% and 66.2%), had a white ethnicity (95.4% and 90.4%), 494 participants (32.3%) and 541 participants (33.3%) were obese, and the mean ages were 59.92 (s.d. 12.94) and 58.77 (s.d. 13.42) (Table 1). In terms of clinical characteristics, 76% fulfilled the 1987 ACR criteria in both samples, mean symptom duration was 2.2 years (s.d. 4.7 and 4.6 for HAQ-DI and DAS28 samples, respectively), mean MTX start dose was 12.1 (s.d. 3.0) mg/week for both samples and 28.4% and 28.5% had two or more comorbidities in the HAQ-DI and DAS28 samples, respectively.

**Table 1.** Baseline characteristics of the sample for the analysis of HAQ-DI (*N* = 1529) and DAS28 (*n* = 1626)

Characteristics	Frequencies (%)/mean (s.d.)			
	HAQ-DI sample (*N* = 1529)	Missing	DAS28 sample (*N* = 1626)	Missing
Demographic and lifestyle factors				
Age, years	59.92 (12.94)	0 (0%)	58.77 (13.42)	0 (0%)
Gender, female	1025 (67.0%)	0 (0%)	1076 (66.2%)	0 (0%)
Ethnicity, white	1458 (95.4%)	13 (0.9%)	1470 (90.4%)	97 (5.9%)
BMI, kg/m²	28.25 (5.96)	0 (0%)	28.37 (6.04)	0 (0%)
BMI categories^a^				
Underweight	16 (1.0%)		22 (1.4%)	
Normal weight	492 (32.2%)		505 (31.1%)	
Overweight	527 (34.5%)		558 (34.3%)	
Obesity	494 (32.3%)		541 (33.3%)	
Alcohol intake, yes	1055 (69.0%)	26 (1.7%)	1108 (68.1%)	24 (1.5%)
IMD fifths:		55 (3.6%)		64 (3.9%)
1: most deprived	164 (10.7%)		181 (11.1%)	
2	263 (17.2%)		285 (17.5%)	
3	295 (19.3%)		318 (19.6%)	
4	371 (24.3%)		384 (23.6%)	
5: least deprived	381 (24.9%)		394 (24.2%)	
Smoking status		6 (0.4%)	645 (39.7%)	7 (0.4%)
Never	627 (41.0%)			
Former	249 (16.3%)		308 (18.9%)	
Current	647 (42.3%)		666 (41.0%)	
Physical activity		10 (0.7%)	67 (4.1%)	91 (5.5%)
Much more	74 (4.8%)			
More	246 (16.1%)		234 (14.4%)	
The same	381 (24.9%)		378 (23.2%)	
Less	544 (35.6%)		559 (34.4%)	
Much less	274 (17.9%)		297 (18.3%)	
Clinical factors				
Fulfilled 1987 ACR criteria	1163 (76.0%)	133 (8.7%)	1236 (76.0%)	130 (7.9%)
Symptom duration, years	2.23 (4.70)	141 (9.2%)	2.18 (4.55)	153 (9.4%)
MTX starting dose, mg/week	12.09 (2.99)	17 (1.1%)	12.11 (2.99)	15 (0.9%)
Comorbidities, two or more	434 (28.4%)	0 (0.0%)	463 (28.5%)	0 (0.0%)
HAQ-DI score (0–3)	1.07 (0.73)	0 (0%)	1.09 (0.74)	92 (5.7%)
DAS28-CRP (0.96–10)	4.16 (1.34)	65 (4.3%)	4.23 (1.34)	0 (0%)
Tender joint count (0*–*28)	7.57 (7.37)	43 (2.8%)	7.96 (7.56)	0 (0%)
Swollen joint count (0*–*28)	6.01 (5.57)	45 (2.9%)	6.18 (5.71)	0 (0%)
CRP value, mg/l	14.36 (23.50)	13 (0.9%)	14.32 (23.13)	0 (0%)
VAS general wellbeing (0*–*100 mm)	40.4 (23.7)	8 (0.5%)	41.8 (23.7)	0 (0%)

a BMI categories defined as: underweight (BMI <18.5 kg/m^2^), normal weight (BMI 18.5–24.9 kg/m^2^), overweight (BMI 25.0–29.9 kg/m^2^), obesity (BMI ≥30.0 kg/m^2^). DAS28: 28-joint DAS; HAQ-DI, HAQ—Disability Index; IMD: Index of Multiple Deprivation; VAS: visual analogue scale.

### The relationship between area-level deprivation and HAQ-DI and DAS28 scores

Those living in the most deprived areas were more likely to have higher self-reported disability scores (measured through HAQ-DI) [adjusted (adj) regression coefficient 0.32 (95% CI 0.19, 0.45)] and DAS (measured through DAS28) [adj regression coefficient 0.34 (95% CI 0.11, 0.58)] over the subsequent year, compared with those living in the least deprived areas (Table 2). Stratified analyses indicated that the relationship between higher deprivation and DAS28 was stronger for obese *vs* non-obese people with RA [adj regression coefficients 0.39 (95% CI 0.02, 0.76) for obese people and 0.22 (95% CI –0.09, 0.52) for non-obese people] (Table 3). Out of the different components of DAS28, area-level deprivation was only associated with more tender joints and higher VAS general wellbeing score (Supplementary Table S1, available at *Rheumatology* online).

**Table 2.** Linear mixed effect models for the relationships of deprivation and obesity with HAQ-DI and DAS28 score

	HAQ-DI score (0–3)		DAS28 score (0.96–10)	
	Regression coefficient (95% CI)		Regression coefficient (95% CI)	
	Unadjusted	Adjusted	Unadjusted	Adjusted
IMD quintiles				
1: most deprived	**0.38 (0.25, 0.50)**	**0.32 (0.19, 0.45)**	**0.51 (0.31, 0.71)**	**0.34 (0.11, 0.58)**
2	**0.18 (0.07, 0.28)**	**0.14 (0.03, 0.25)**	**0.36 (0.19, 0.52)**	**0.30 (0.09, 0.51)**
3	0.02 (–0.08, 0.12)	0.03 (–0.07, 0.14)	0.16 (–0.01, 0.32)	0.15 (–0.06, 0.35)
4	–0.06 (–0.16, 0.03)	**–0.12 (–0.22, –0.03)**	0.03 (–0.13, 0.18)	–0.01 (–0.19, 0.17)
5: least deprived	ref	ref	ref	ref
Obesity				
Obesity	**0.19 (0.13, 0.24)**	**0.13 (0.06, 0.20)**	**0.41 (0.30, 0.51)**	**0.17 (0.04, 0.31)**
Non-obesity	ref	ref	ref	ref
BMI per 1 kg/m^2^ increment	**0.02 (0.02, 0.03)**	**0.01 (0.00, 0.01)**	**0.04 (0.03, 0.05)**	**0.01 (0.00, 0.02)**

Obesity analyses adjusted for age, gender, ethnicity, deprivation, smoking, physical activity and alcohol consumption. Socioeconomic position analyses adjusted for age and gender. Bold values indicate statistical significance. For HAQ-DI, no interaction between obesity and gender (*P* = 0.273), obesity and IMD (*P* = 0.188), or IMD and gender (*P* = 0.909). For DAS28, evidence of interaction between obesity and IMD (*P* = 0.020), but not for obesity and gender (*P* = 0.676), or IMD and gender (*P* = 0.377). DAS28: 28-joint DAS; HAQ-DI: HAQ—Disability Index; IMD: Index of Multiple Deprivation.

**Table 3.** Linear mixed effect models for the relationship between deprivation and HAQ-DI and DAS28 score by obesity status

	HAQ-DI score (0–3)		DAS28 score (0–10)	
	Regression coefficient (95% CI)		Regression coefficient (95% CI)	
	No obesity	Obesity	No obesity	Obesity
IMD quintiles				
1: most deprived	**0.31 (0.14, 0.47)**	**0.25 (0.04, 0.45)**	0.22 (–0.09, 0.52)	**0.39 (0.02, 0.76)**
2	0.09 (–0.04, 0.22)	0.17 (–0.02, 0.37)	0.15 (–0.10, 0.40)	**0.49 (0.14, 0.83)**
3	0.01 (–0.12, 0.13)	0.07 (–0.13, 0.26)	0.19 (–0.05, 0.43)	0.02 (–0.32, 0.37)
4	–0.10 (–0.22, 0.01)	–0.15(–0.34, 0.04)	0.06 (–0.16, 0.28)	–0.17 (–0.51, 0.17)
5: least deprived	ref	ref	ref	ref

Adjusted for age and gender. Bold values indicate statistical significance. DAS28: 28-joint DAS; HAQ-DI: HAQ—Disability Index; IMD: Index of Multiple Deprivation.

### The relationship between obesity and HAQ-DI and DAS28 scores

Over time, obese people with RA at baseline were more likely to have higher HAQ-DI scores [adj regression coefficient 0.13 (95% CI 0.06, 0.20)] and DAS28 scores [adj regression coefficient 0.17 (95% CI 0.04, 0.31)] over the subsequent year, compared with non-obese people with RA (Table 2). A 1-unit BMI increment was also associated with a 0.01 (95% CI 0.00, 0.01) increase in HAQ-DI score and a 0.01 (95% CI 0.00, 0.02) increase in DAS28 score. Stratified analyses indicated that the relationship between obesity and DAS28 was dependent on gender: adj regression coefficients 0.14 (95% CI –0.11, 0.39) for men and 0.20 (95% CI 0.03, 0.36) for women (Table 4). However, no substantial gender differences were observed for the different components of the DAS28 (Supplementary Table S2, available at *Rheumatology* online).

**Table 4.** Linear mixed effect models for the relationship between obesity and DAS28 score by gender

	HAQ-DI score (0–3)		DAS28 score (0–10)	
	Regression coefficient (95% CI)		Regression coefficient (95% CI)	
	Men	Women	Men	Women
Obesity				
Obesity	**0.18 (0.06, 0.30)**	**0.11 (0.03, 0.19)**	0.14 (–0.11, 0.39)	**0.20 (0.03, 0.36)**
Non-obesity	ref	ref	ref	ref
BMI per 1 kg/m^2^ increment	0.01 (–0.00, 0.02)	**0.01 (0.00, 0.02)**	0.01 (–0.02, 0.03)	**0.01 (0.00, 0.02)**

Adjusted for age, ethnicity, deprivation, smoking, physical activity and alcohol consumption. Bold values indicate statistical significance. DAS28: 28-joint DAS.

The mediating effect of BMI for the relationship between area-level deprivation and HAQ-DI and DAS28 scores.

BMI mediated part of the association between higher deprivation and HAQ-DI scores (14.24%) and DAS28 scores (17.26%) in the total study population (men and women combined). However, there were no indirect effects when restricting the sample to men only. For women, the mediating effect of BMI were 17.79% and 25.56% for HAQ-DI and DAS28, respectively (Table 5).

**Table 5.** The total, direct and indirect effect (via BMI) of area-level deprivation on average HAQ-DI and DAS28 scores

	β-coefficient (95% CI)			Proportion mediated (95% CI)^a^
	Total	Direct	Indirect	
Average HAQ-DI score (0–3)				
Total	**0.097 (0.067, 0.120)**	**0.083 (0.054, 0.110)**	**0.014 (0.007, 0.020)**	14.24% (7.77%, 23.00%)
Men	**0.098 (0.049, 0.145)**	**0.091 (0.042, 0.136)**	0.007 (–0.001, 0.019)	
Women	**0.095 (0.065, 0.130)**	**0.078 (0.050, 0.110)**	**0.017 (0.008, 0.030)**	17.79% (8.67%, 30.00%)
Average DAS28 score (0–10)				
Total	**0.122 (0.083, 0.162)**	**0.101 (0.062, 0.140)**	**0.021 (0.012, 0.032)**	17.26% (9.72%, 29.00%)
Men	**0.123 (0.055, 0.190)**	**0.119 (0.051, 0.190)**	0.004 (–0.002, 0.020)	
Women	**0.120 (0.007, 0.170)**	**0.089 (0.039, 0.130)**	**0.031 (0.018, 0.046)**	25.56% (14.35%, 44.00%)

a Calculated by indirect effect/total effect × 100%. 95% CI estimated with bootstrapping. For men, there were no indirect effects so the proportion mediated was not calculated. The exposure–mediator interaction was non-significant for both HAQ-DI (*P* = 0.83) and DAS28 (*P* = 0.09), indicating that the no exposure–mediator interaction assumption holds. Bold values indicate statistical significance. DAS28: 28-joint DAS; HAQ-DI: HAQ—Disability Index.

Results from the sensitivity analysis to test sequential ignorability indicated that the degree of unmeasured confounding required to explain way the observed mediation effect for both HAQ-DI and DAS28 was a ρ of 0.2.

## Discussion

In this study of adults with RA starting MTX for the first time, we found that area-level deprivation was associated with worse disability and disease activity over the subsequent year. We found that a proportion of these associations could be explained by obesity.

The temporal relationship between a lower SEP (measured through both individual indicators and area-level measures) and worse outcomes in RA has been found previously [6–9]. However, longitudinal studies performed in England are limited. Given the complex interactions between SEP and obesity, it was important to investigate the interactions between area-level deprivation and obesity on RA disease outcomes. We found that the association between deprivation and DAS28 was stronger among those with obesity *vs* those without, indicating that in people who live in more deprived areas, having obesity is associated with worse disease outcomes. We further found that part of the association between deprivation and RA outcomes can be explained by BMI. Notably, when restricting the sample to men or women only, the mediating effect of BMI was only observed among women for both disability and disease activity. This may partly be explained by the stronger relationship between lower SEP and obesity among women compared with men in the general population [11]. Another explanation may be that we found that the association between obesity and DAS28 was stronger among women than men; however, no gender differences were observed for the separate components of DAS28. It is therefore also possible that our sample size for men was too small to find an effect. It is also possible that FM may play a role; obesity is associated with FM [27], it is generally more common among women [28] and it has been associated with worse RA outcomes [29]. However, this needs further investigation. Gender differences for the relationship between obesity and RA outcomes have not been studied extensively; however, a Swedish clinical trial assessing MTX also found that obese women were less likely to achieve remission compared with non-obese women or obese men [30]. These gender differences for the relationship between BMI and functional limitations have also been found in the general population [31]. Although the exact reasons for this are unclear, a potential explanation is that men are more likely to underreport limitations whereas women are more willing to report, or even overestimate, their physical limitations [32].

In general, a large part of the association between area-level deprivation and worse RA outcomes could not be explained by obesity, indicating that other factors may be important too. For example, it has been suggested that differences in disease progression could be due to lower patient participation (i.e. less rheumatologist visits) [33] and treatment delays [34] in people with RA with lower SEP. Potentially this may lead to people with RA missing the ‘window of opportunity’ in the early stages of disease [35], resulting in worse outcomes over time among those with a lower SEP [36].

The relationship between obesity and worse RA outcomes has been reported in previous studies [12–16]. Although it is uncertain what the exact mechanisms for this relationship are, there are a few potential explanations. Firstly, inflammation and immunological changes instigated by adipose tissue may drive disease activity [18]; however, in our study we did not find an association between obesity and CRP levels in people with RA. Secondly, obese people with RA may be less responsive to rheumatic medications, including MTX, and therefore have higher disease activity than those without obesity [37]. It has been hypothesized that this may also be due to higher levels of pro-inflammatory cytokines in obese individuals [37]. Thirdly, self-reported musculoskeletal pain is higher in obese people with RA [38], which may be partly explained by disrupted neurotransmitters and hormones [27]. In line with this third point, we found that the higher DAS28 scores in obese individuals were driven by the subjective components, tender joint count and VAS general wellbeing, rather than swollen joint counts or CRP levels. Higher pain may further impact daily activities in the HAQ-DI.

Strengths of this study include that it is a prospective cohort study with measurements of HAQ-DI and DAS28 at two or more time points, allowing the analysis of temporal associations between deprivation, obesity and RA outcomes. Unfortunately, we only had data about area-level deprivation which we used as a proxy for SEP. Area-level deprivation measures have sometimes been criticized as they misclassify people who experience deprivation but do not live in deprived areas [39]. Therefore, these results need to be validated in future studies where individual-based indicators, such as education, occupation, income or wealth, are used. Although educational level was recorded in RAMS, 41% of the sample had missing values; hence, it was decided to not include this in our analyses. Furthermore, BMI is an imperfect measure of adiposity [40]. It would have been interesting to investigate waist circumference, as waist circumference has a stronger association with inflammatory factors than BMI [41] which may contribute to worse progression of disease. Unfortunately, waist circumference was not recorded in RAMS. RAMS has a short follow-up (max 12 months), which may be too short to investigate the longitudinal effects of socioeconomic factors and obesity on RA disease progression. Lastly, the criteria for selecting the study samples may have resulted in selection bias. The DAS28 sample is slightly larger than the HAQ-DI sample, as the DAS28 components were measured during the CRF and HAQ-DI components were recorded in the patient questionnaire which required the additional steps of completing it and sending it to the co-ordinating research centre by the study nurse or the participant. However, baseline characteristics between the two groups did not differ substantially, except in terms of ethnicity (Table 1). Moreover, loss to follow-up was differential (Supplementary Table S3, available at *Rheumatology* online, shows the characteristics of people who were lost to follow-up); those from more deprived areas were more likely to not have at least one follow-up measurement and therefore to be excluded from this study. In addition to this, people in disadvantaged groups are less likely to participate in research generally [42]. Therefore, it is possible that our study population may not represent the whole RA population in England.

With these limitations in mind, there are some important implications of the findings of this study. We cannot definitely conclude that the relationships found in this study are causal due to the observational nature of our study; however, we did find that obesity is an important factor for social disparities in RA outcomes. Recently updated NICE guidelines suggest that multicomponent treatment interventions should be the first choice of treatment, which includes behaviour change strategies to improve people’s diet and increase physical activity [43]. If lifestyle interventions are ineffective, medication or bariatric surgery can be considered [43]. Studies assessing the impact of weight loss interventions in people with RA are limited. A retrospective study indicated that weight loss of ≥5 kg was associated with reduced disease activity [44]. More recently, a pilot randomized clinical trial including 50 participants reported that a weight and pain management programme is effective in improving function and reducing pain in obese people with established RA [45]. There is also emerging evidence that disease activity is reduced after bariatric surgery [46–48]. These studies show potential for weight loss interventions to improve RA outcomes. However, it is unknown whether weight loss interventions in obese people with RA are effective in different socioeconomic groups, for which further research is indicated.

To conclude, improving disease outcomes is a key aim for the management of RA. In order to address socioeconomic disparities in RA outcomes, it is important to understand why these discrepancies exist and whether they are modifiable. We found that part of the association between area-level deprivation and both disease activity and functional disability in RA is mediated through obesity. Further research is needed to understand whether weight loss interventions for obese people with RA are effective in lower socioeconomic groups.

## Supplementary Material

keac662_Supplementary_Data

## Data Availability

The data underlying this article cannot be shared publicly to protect the privacy of individuals that participated in the study. The data will be shared on reasonable request to the corresponding author.
