# Supplementary material for: Is the relationship between deprivation and outcomes in rheumatoid arthritis mediated by body mass index? A longitudinal cohort study
Source: Rheumatology (Oxford). 2022 Nov 28;62(7):2394–401. doi: 10.1093/rheumatology/keac662 (PMC10321122; doi:10.1093/rheumatology/keac662)
Supplement: keac662_Supplementary_Data [file keac662_supplementary_data.docx]

**Supplementary File**


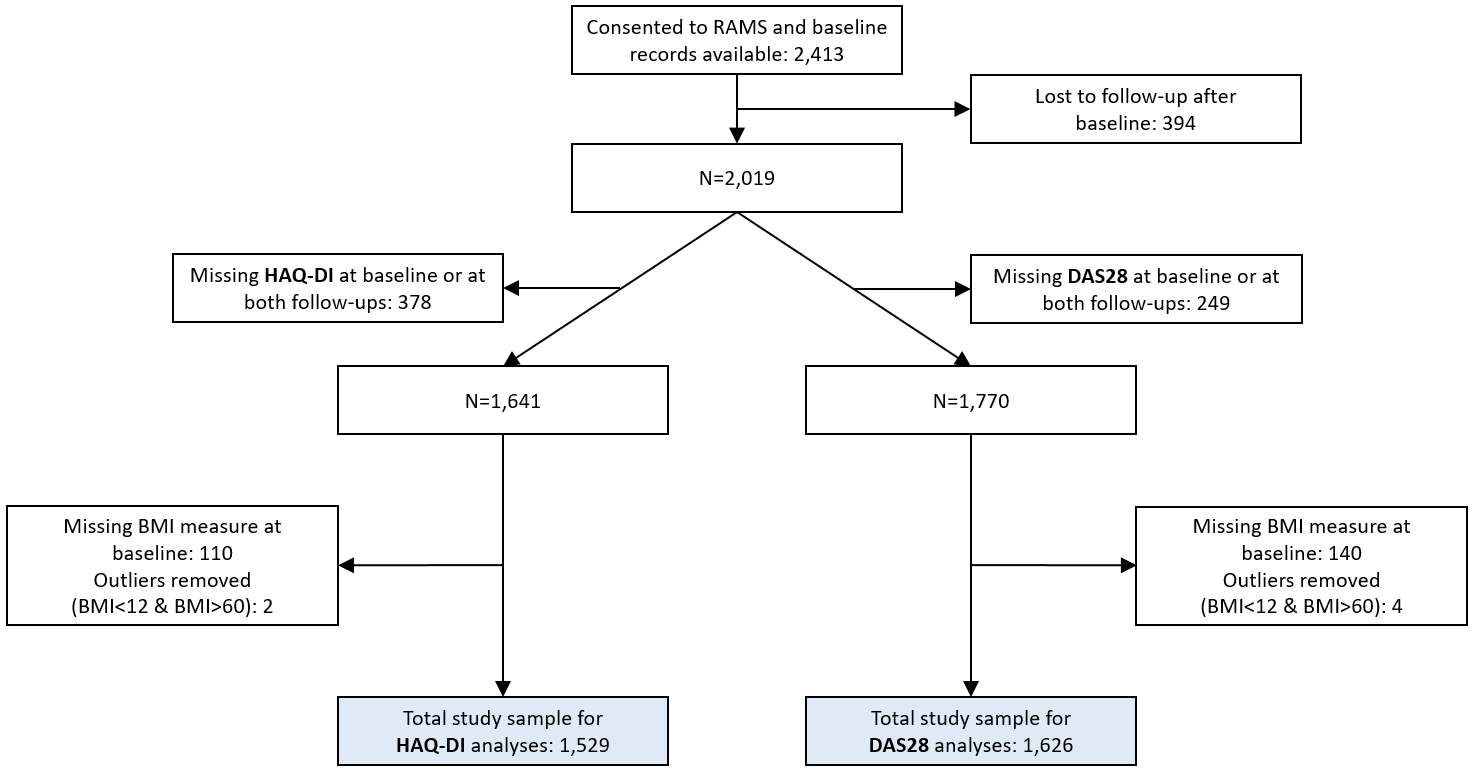


Figure S1: Flowchart of participant selection for HAQ-DI and DAS28 analyses

Table S1: Linear mixed effect models for the relationships of deprivation and obesity with separate DAS28 components (tender joint counts, swollen joint counts, CRP levels and VAS general wellbeing)

|  | Regression coefficient (95% CI) | | | | | | | |
| --- | --- | --- | --- | --- | --- | --- | --- | --- |
|  | **Tender joint count (0–28)** | | **Swollen joint count (0–28)** | | **CRP levels** | | **VAS general wellbeing (0-100)** | |
|  | Unadjusted | Adjusted | Unadjusted | Adjusted | Unadjusted | Adjusted | Unadjusted | Adjusted |
| *Index of Multiple Deprivation quintiles* | | | | | | | | |
| 1: most deprived | **2.31 (1.27, 3.34)** | **2.16 (0.83, 3.49)** | 0.29 (-0.37, 0.95) | -0.23 (-1.25, 0.79) | 1.02 (-1.27, 3.31) | 0.93 (-3.28, 5.14) | **9.51 (6.32, 12.69)** | **7.95 (3.81, 12.09)** |
| 2 | **2.01 (1.12, 2.91)** | **1.57 (0.39, 2.76)** | **0.64 (0.09, 1.19)** | 0.53 (-0.36, 1.42) | -0.42 (-2.41, 1.57) | -0.47 (-4.11, 3.17) | **6.08 (3.37, 8.78)** | **7.07 (3.57, 10.57)** |
| 3 | **1.23 (0.38, 2.09)** | **1.19 (0.10, 2.28)** | 0.24 (-0.30, 0.78) | 0.14 (-0.71, 0.99) | 0.64 (-1.29, 2.57) | 1.85 (-1.70, 5.40) | 1.18 (-1.50, 3.85) | 2.15 (-1.37, 5.67) |
| 4 | 0.17 (-0.64, 0.98) | -0.15 (-1.19, 0.88) | -0.10 (-0.61, 0.40) | -0.49 (-1.29, 0.31) | 0.46 (-1.38, 2.31) | 0.94 (-2.37, 4.24) | 0.16 (-2.36, 2.68) | 1.08 (-2.14, 4.30) |
| 5: least deprived | ref | ref | ref | ref | ref | ref | ref | ref |
| *Obesity* | | | | | | | | |
| Obesity | **1.50 (0.95, 2.04)** | **0.90 (0.12, 1.69)** | **0.65 (0.29, 1.02)** | 0.52 (-0.09, 1.12) | 1.23 (-0.10, 2.57) | -1.41 (-3.94, 1.12) | **5.65 (3.89, 7.42)** | 1.43 (-0.99, 3.84) |
| Non-obesity | ref | ref | ref | ref | ref | ref | ref | ref |
| BMI per 1 kg/m^2^ increment | 0.16 (0.11, 0.20) | 0.07 (0.01, 0.13) | **0.06 (0.03, 0.09)** | 0.03 (-0.01, 0.08) | **0.11 (0.00, 0.21)** | -0.17 (-0.37, 0.03) | **0.47 (0.33, 0.61)** | 0.00 (-0.19, 0.19) |

BMI, body mass index; CI, confidence interval; DAS28, disease activity score 28; kg, kilograms; m, meters. Obesity analyses adjusted for age, gender, ethnicity, deprivation, smoking, physical activity and alcohol consumption. Socioeconomic position analyses adjusted for age and gender. Bold values indicate statistical significance.

Table S2: Linear mixed effect models for the relationships of deprivation and obesity with separate DAS28 components (tender joint counts, swollen joint counts, CRP levels and VAS general wellbeing) by gender

|  | Regression coefficient (95% CI) | | | | | | | |
| --- | --- | --- | --- | --- | --- | --- | --- | --- |
|  | **Tender joint count (0–28)** | | **Swollen joint count (0–28)** | | **CRP levels** | | **VAS general wellbeing (0-100)** | |
|  | Men | Women | Men | Women | Men | Women | Men | Women |
| *Index of Multiple Deprivation quintiles* | | | | | | | | |
| 1: most deprived | 1.81 (-0.42, 4.03) | **2.14 (0.50, 3.78)** | -0.56 (-2.46, 1.35) | -0.25 (-1.45, 0.95) | 4.51 (-3.65, | -0.33 (-5.43, 4.77) | **11.17 (3.82, 18.51)** | **6.62 (1.70, 11.54)** |
| 2 | **3.00 (1.17, 4.83)** | 0.79 (-0.67, 2.25) | 1.17 (-0.37, 2.71) | 0.11 (-0.95, 1.17) | -3.28 (-9.83, 3.28) | 0.83 (-3.59, 5.25) | **7.84 (1.98, 13.70)** | **6.47 (2.11, 10.82)** |
| 3 | 0.74 (-0.94, 2.42) | **1.43 (0.00, 2.85)** | -0.16 (-1.60, 1.29) | 0.31 (-0.72, 1.35) | 2.24 (-3.91, 8.40) | 1.65 (-2.62, 5.93) | -0.21 (-5.89, 5.47) | 3.50 (-0.83, 7.82) |
| 4 | -0.32 (-1.90, 1.27) | -0.15 (-1.52, 1.21) | -0.57 (-1.93, 0.79) | -0.53 (-1.54, 0.47) | -0.39 (-6.22, 5.45) | 1.61 (-2.50, 5.73) | 4.98 (-0.30, 10.27) | -0.99 (-5.10, 3.13) |
| 5: least deprived | ref | ref | ref | ref | ref | ref | ref | ref |
| *Obesity* | | | | | | | | |
| Obesity | 1.03 (-0.21, 2.27) | 0.84 (-0.16, 1.84) | 0.55 (-0.53, 1.63) | 0.53 (-0.23, 1.29) | -3.68 (-8.22, 0.86) | 0.33 (-2.68, 3.33) | 3.36 (-0.83, 7.55) | 0.58 (-2.38, 3.54) |
| Non-obesity | ref | ref | ref | ref | ref | ref | ref | ref |
| BMI per 1 kg/m^2^ increment | 0.06 (-0.05, 0.18) | 0.07 (-0.00, 0.15) | 0.02 (-0.08, 0.12) | 0.04 (-0.02, 0.10) | -0.64 (-1.07, -0.21) | -0.01 (-0.23, 0.22) | 0.27 (-0.11, 0.65) | -0.07 (-0.29, 0.14) |

BMI, body mass index; CI, confidence interval; DAS28, disease activity score 28; kg, kilograms; m, meters. Obesity analyses adjusted for age, gender, ethnicity, deprivation, smoking, physical activity and alcohol consumption. Socioeconomic position analyses adjusted for age and gender. Bold values indicate statistical significance.

Table S3: Characteristics of people who were excluded due to loss to follow-up in both samples (N=394) and who had missing BMI the HAQ-DI analyses (N=112)

| Characteristics | Frequencies (%) / mean (SD) | | | |
| --- | --- | --- | --- | --- |
|  | Excluded due to lost to follow-up after baseline (N=394) | Missing | Excluded due to missing BMI values for the HAQ-DI analyses (N=112) | Missing |
| Age, *years* | 56.45 (13.99) | 6 (1.5%) | 60.94 (12.13) | 0 (0.0%) |
| Gender, *female* | 268 (68.0%) | 6 (1.5%) | 77 (68.8%) | 0 (0.0%) |
| Ethnicity, *white* | 268 (68.0%) | 101 (25.6%) | 105 (93.8%) | 1 (0.9%) |
| IMD fifths:  *1: most deprived* | 69 (17.5%) | 18 (4.6%) | 14 (12.5%) | 5 (4.5%) |
| *2* | 77 (19.5%) |  | 22 (19.6%) |  |
| *3* | 74 (18.8%) |  | 26 (23.2%) |  |
| *4* | 85 (21.6%) |  | 19 (17.0%) |  |
| *5: least deprived* | 71 (18.0%) |  | 26 (23.2%) |  |
| Smoking status:  *Never* | 114 (28.9%) | 16 (4.1%) | 44 (39.3%) | 6 (5.4%) |
| *Former* | 146 (37.1%) |  | 39 (34.8%) |  |
| *Current* | 118 (29.9%) |  | 23 (20.5%) |  |
| Physical activity:  *Much more* | 16 (4.1%) | 102 (25.9%) | 8 (7.1%) | 0 (0.0%) |
| *More* | 37 (9.4%) |  | 18 (16.1%) |  |
| *The same* | 75 (19.0%) |  | 35 (31.3%) |  |
| *Less* | 102 (25.9%) |  | 36 (32.1%) |  |
| *Much less* | 62 (15.7%) |  | 15 (13.4%) |  |
| Alcohol intake, *Yes* | 236 (59.9%) | 23 (5.8%) | 76 (67.9%) | 11 (9.8%) |
| BMI, kg/m² | 28.18 (6.24) | 61 (15.5%) | - | - |
| BMI categories†: |  |  |  |  |
| *Underweight* | 10 (2.5%) |  |  |  |
| *Normal weight* | 102 (25.9%) |  |  |  |
| *Overweight* | 114 (28.9%) |  |  |  |
| *Obesity* | 107 (27.2%) |  |  |  |

BMI, body mass index; HAQ-DI, health assessment questionnaire – disability index; IMD, index of multiple deprivation; kg, kilograms; m, meters; SD, standard deviation. †BMI categories defined as: underweight (BMI <18.5 kg/m^2^), normal weight (BMI 18.5–24.9 kg/m^2^), overweight (BMI 25.0–29.9 kg/m^2^), obesity (BMI ≥ 30.0 kg/m^2^).
